# Significant Association of Variable Number Tandem Repeat Polymorphism rs58335419 in the MIR137 Gene With the Risk of Gastric and Colon Cancers

**DOI:** 10.3389/bjbs.2021.10095

**Published:** 2022-02-04

**Authors:** Pegah Jafari, Sedighe Baghernia, Mehdi Moghanibashi, Parisa Mohamadynejad

**Affiliations:** ^1^ Department of Biology, Faculty of Basic Sciences, Kazerun Branch, Islamic Azad University, Kazerun, Iran; ^2^ Department of Genetics, School of Medicine, Kazerun Branch, Islamic Azad University, Kazerun, Iran; ^3^ Department of Biology, Faculty of Basic Sciences, Shahrekord Branch, Islamic Azad University, Shahrekord, Iran

**Keywords:** MIR137 gene, VNTR, rs58335419, gastric cancer, colon cancer

## Abstract

**The purpose of the article:** The MIR137 gene acts as a tumor-suppressor gene in colon and gastric cancers. The aim of this study was to investigate the association of functional variable number tandem repeat (VNTR) polymorphism rs58335419 locating in the upstream of the MIR137 gene with the risk of colon and gastric cancers.

**Materials and methods:** Totally, 429 individuals were contributed in the study, including 154 colon and 120 gastric cancer patients and 155 healthy controls. The target VNTR was genotyped using PCR and electrophoresis for all samples. Statistical analysis was performed using SPSS 21.0 software and by T, χ2 and logistic regression tests.

**Results:** Excluding the rare genotypes, our results showed that genotype 3/5 (95% CI = 1.08–3.73, OR = 2.01, *p* = 0.026) significantly increased the risk of colon cancer but not gastric cancer (95% CI = 0.88–3.30, OR = 1.70, *p* = 0.114). Also, in the stratification analysis for VNTRs and sex, genotypes 3/4 (95% CI = 1.00–6.07, OR = 2.46, *p* = 0.049) and 3/5 (95% CI = 1.25–7.18, OR = 2.99, *p* = 0.014) significantly increased the risk of colon cancer in men but not in women. In addition, all genotypes including the rare genotypes as a group, significantly increase the risk of gastric (95% CI = 1.14–3.00, OR = 1.85, *p* = 0.012) and colon (95% CI = 1.38–3.43, OR = 2.17, *p* = 0.001) cancers compared to the genotype 3/3 as a reference.

**Conclusion:** The results show that increasing the copy of VNTR in the MIR137 gene, increases the risk of colon and gastric cancers and can serve as a marker for susceptibility to colon and gastric cancers.

## Introduction

MicroRNAs (miRNAs), are small non-coding RNAs about 22 nucleotides in length that play a key role in the regulation of post-transcriptional level (1, 2). Bioinformatics studies have shown that up to 80% of genes are regulated by miRNAs (3).

Despite its important role in brain function (4) and its association with the causes of many psychiatric disorders including schizophrenia (5) and bipolar disorder (6–8), the MIR-137 gene (chromosome 1p22) has been shown to contribute to various cancers including gastric and colon carcinoma (9, 10).

Hundreds of genes have been identified that are affected by miR-137 (50 genes have been showed to be directly regulated by miR-137), and target genes are involved in various biological pathways including cell cycle, proliferation and differentiation (3, 11).

Studies have shown that miR-137 act as a tumor suppressor in colon cancer by negatively regulating a range of downstream targets (9, 12). The Wnt and Notch signaling pathways, which are involved in colorectal cancer progression, are inhibited by miR-137 through the MSI1 gene (13).

In addition, miR-137 is involved in gastric cancer by acting as a tumor suppressor gene (14, 15). MiR-137 downregulation especially by epigenetic process has been indicated in gastric cancer, leading to overexpression of Cdc42 as an oncogene (16).

The human genome contains millions of repetitive sequences, some of which are tandem and variable in number of repeating units called variable number tandem repeats (VNTRs). VNTRs represent one of the sources of polymorphisms in the human genome, and some of them are located in coding, untranslated or regulatory regions of genes. Copy number variation in some VNTRs has a significant functional impact and can be related to gene expression (17).

One of the polymorphism in the MIR137 gene is a VNTR polymorphism rs58335419 (15 base pairs in length for each repetitive unit) located in the six bases in upstream of the pre-miR-137 transcript (18). Several studies show that there is a wild-type variant with three repeats, the shortest known length, and two relatively common minor alleles with 4 and 5 repeats (10, 19).

Previous studies have shown that VNTR polymorphism rs58335419 can be functional as the increase in VNTR length is associated with a significant positive del-miR-137 transcripts that affects gene expression by alternative splicing (19). Various allele in this VNTR can also affect reporter gene expression (20) and alteration in the number of tandem repeats in this VNTR interferes with the mature processing and function of miR-137 (18).

Given miR-137 downregulation in gastric and colon cancers and the functional role of VNTR polymorphism rs58335419 in MIR-137 expression, for the first time we analyzed the association between the length of VNTR of MIR137 gene and the risk of gastric and colon cancers.

## Materials and Methods

In this study, whole blood samples were taken from 154 (with age range 30–83 years and mean 57.32 ± 11.65 years) and 120 (with age range 26–85 years and mean 58.79 ± 12.06 years) patients with colon and gastric cancer, respectively, all of whom were diagnosed histologically in the Pathology Department of Namazi Hospital (Shiraz, Iran). In addition, 155 (with age range 26–91 years and mean 60.36 ± 15.39 years) healthy blood donor volunteer from the same geographic area were participated as control group without clinical evidence and family history. All of patients and controls were unrelated Iranian.

The Institutional Review Board of the Islamic Azad University, Kazerun Branch approved the study protocol and the informed consent form was obtained from the patients participating in this study.

DNA extraction was performed using GTP Kit (Gene Transfer Pioneer, IRAN), then a pair of primers (F: 5-CCC​GAG​GAA​ATG​AAA​AGA​AC-3 and R: 5- TTG​GGC​AGG​AAG​CAG​CCG​AG-3) were designed using OLIGO software (version 7) based on the sequences available on the NCBI to determine the length of the VNTR rs58335419 in the miR-137 gene. The primer sequences were checked through BLAST software on the NCBI site to ensure the specificities of primers.

Optimized PCR was carried out in a single tube containing 25 μL of reaction with 12.5 μL master mix PCR (Ampliqon), 1.5 μL DNA (50–100 ng) of genomic DNA, 9 μL of distilled water, 1 μL of each primer (10 pmol). PCR was performed at 94°C for 10 min for initial denaturation, followed by 30 cycles of denaturation at 94°C for 1 min, annealing at temperature 62°C for 45 s, extension at 72°C for 45 s and final extension at 72°C for 10 min. Finally, the PCR products were separated by 3% agarose gel at 120 v for 30 min, followed by safe staining instead of ethidium bromide and photographed under Gel Documentation system.

## Statistical Analysis

T test was used to compare differences in demographic variables between patients and the normal group. Unconditional logistic regression models were used to evaluate the associations between the VNTR genotype and risk of gastric and colon cancers by calculating the odds ratio (OR) and 95% confidence interval (CI). Statistical analysis was performed using IBM SPSS 21 software and probability level was 0.05.

## Results

**FIGURE 1:**
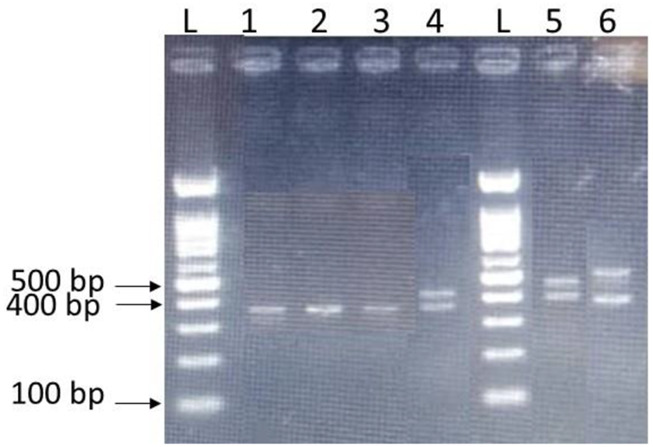
Genotyping of rs58335419 VNTR polymorphism by PCR and gel electrophoresis on 3% agarose gel. Genotype 3/4 results in two bands 396 bp and 411 bp (lane 4), genotype 3/5 results two bands 396 bp and 426 bp (lane 5), genotype 3/11 results to 396 bp and 536 bp (lane 6), and genotype 3/3 (396 bp, lane 1, 2, and 3). Lane L is the 100 bp DNA standard marker.

The clinical and demographic data of cases and controls are summarized in Table 1. A specific primer pair was used to amplify the polymorphic region of VNTR in the miRNA-137 gene. The amplified fragment for the allele with three repeats is 396 bp and 15 bp is added to the length of the amplifying fragment for each repeat. To determine the accuracy of PCR, the product was electrophoresed on 3% agarose gel (Figure 1).

**TABLE 1:** General characteristics of cases and controls.

Variable	Categories	Controls	Gastric cancer patients	*p*	Colon cancer patients	*p*
Number		155	120		154	
Age of onset (years)	Age range	26–91	26–85		30–83	
	Mean ± SD	60.36 ± 15.39	58.79 ± 12.06	0.19	57.32 ± 11.65	0.59
Sex	Male	59.96 ± 14.65	59.79 ± 11.54	0.62	56.85 ± 12.12	0.15
	Female	60.90 ± 16.45	56 ± 32 ± 13.11	0.11	57.84 ± 11.20	0.21
Type of cancer	Intestinal		51			
	Diffuse		25			
	Unknown		44			
Metastasis	No				132	
	Yes				22	

Distribution and frequencies of different genotypes of VNTR rs58335419 polymorphism that were identified in the patients and control groups are listed in Table 2. Most of the genotypes identified in the present study were rare in control and patient groups, so they were excluded and only genotypes 3/3, 3/4, and 3/5 were analyzed (Table 3). In this way, genotype 3/5 (95% CI = 1.08–3.73, OR = 2.01, *p* = 0.026) but not genotype 3/4 (*p* = 0.135) significantly increased the risk of colon cancer compared to 3/3 as a reference genotype (Table 3). In the same analysis for gastric cancer, none of the genotypes were associated with disease risk (Table 3).

**TABLE 2:** Frequency of different genotypes of VNTR rs58335419 polymorphisms in MIR-137 gene in colon and gastric cancers in patients and controls.

Genotypes	4/10	3/3	3/4	3/5	3/12	4/5	5/5	3/6	4/4	3/10	3/11	Total
Controls	2 (1.3%)	87 (56.1%)	22 (14.2%)	25 (16.1%)	2 (1.3%)	6 (3.9%)	3 (1.9%)	3 (1.9%)	1 (0.6%)	1 (0.6%)	3 (1.9%)	155 (100.0%)
GC patients	4 (3.3%)	49 (40.8%)	17 (14.2%)	24 (20.0%)	3 (2.5%)	6 (5.0%)	4 (3.3%)	8 (6.7%)	2 (1.7%)	3 (2.5%)	0 (0.0%)	120 (100.0%)
CC patients	6 (3.9%)	57 (37.0%)	24 (15.6%)	33 (21.4%)	4 (2.6%)	7 (4.5%)	6 (3.9%)	10 (6.5%)	3 (1.9%)	4 (2.6%)	0 (0.0%)	154 (100.0%)

GC, gastric cancer; CC, colon cancer.

**TABLE 3:** Association between VNTR rs58335419 polymorphisms in MIR-137 gene with the risk of colon and gastric cancers.

Genotypes	3/3 (%)	3/4 (%)	3/5 (%)
Controls	87 (64.9)	22 (16.4)	25 (18.7)
GC patients	49 (54.4)	17 (18.9)	24 (26.7)
Logistic regression test	Reference	*p* = 0.391	*p* = 0.114
		OR = 1.37	OR = 1.70
		CI = 0.66–2.82	CI = 0.88–3.30
CC patients	57 (50.0)	24 (21.1)	33 (28.9)
Logistic regression test	Reference	*p* = 0.135	***p* = 0.026**
		OR = 1.66	OR = 2.01
		CI = 0.85–3.24	CI = 1.08–3.73

GC, gastric cancer; CC, colon cancer. Bold values indicates significant data, where p<0.05.

In the stratification analysis for VNTR polymorphism and sex, genotypes 3/4 (95% CI = 1.00–6.07, OR = 2.46, *p* = 0.049) and 3/5 (95% CI = 1.25–7.18, OR = 2.99, *p* = 0.014) significantly increased the risk of colon cancer in men but not in women. However, none of the genotypes were associated with the risk of gastric cancer in both sexes (genotype 3/5 increased the risk of gastric cancer in men significantly border line, *p* = 0.055).

In a separate analysis, all genotypes (including the rare genotypes that were omitted in the previous analysis) were compared as a group with the genotype 3/3 as a reference, and our analysis showed that these genotypes significantly increase the risk of gastric (95% CI = 1.14–3.00, OR = 1.85, *p* = 0.012) and colon (95% CI = 1.38–3.43, OR = 2.17, *p* = 0.001) cancers.

In stratification analysis for VNTR polymorphism and sex, other genotypes significantly increase the risk of colon cancer (*p* < 0.001) and gastric cancer (*p* = 0.009) in men but not in women. However, none of genotypes are associated with the risk of gastric cancer in men and women compared to genotype 3/3.

In addition, the results of the analysis showed that genotypes 3/4 (*p* = 0.998) and 3/5 (*p* = 0.484) were not statistically significant with the risk of metastasis in colon cancer. Other genotypes increase the risk of metastasis in patients with colon cancer compared to genotype 3/3. Also, the risk of intestinal and diffuse types of gastric cancer were not increased significantly with the 3/4 (*p* = 0.912) and 3/5 (*p* = 0.867) genotypes of VNTR polymorphism.

Other alleles (all alleles except allele with three repeats were in the same group) significantly increased the risk of colon (95% CI = 1.28–2.55, OR = 1.81, *p* = 0.001) and gastric cancers (95% CI = 1.07–2.20, OR = 1.53, *p* = 0.019) compared to allele with three repeats.

Finally, all patients, including gastric and colon cancers as a group were compared to control group (genotype 3/3 as reference) and our analysis showed that the genotype 3/5 significantly increase the risk of cancer (95% CI = 1.08–3.24, OR = 1.87, *p* = 0.025) but not genotype 3/4.

## Discussion

Many studies have shown that a miRNA can affect hundreds of different genes, so that each miRNA can bind to different mRNAs and affect gene expression by degradation or inhibiting mRNA translation (21). MIR 137 is one of the micro RNAs that is involved in some diseases, including cancers (22, 23).

Previous studies have shown that a 15-nucleotide VNTR (rs58335419) is located in the vicinity of the miR-137 gene, six base pairs upstream of the mature miR-137, which regulates its expression (18, 20). So far, several alleles have been identified at this locus but most alleles are rare in the population, however alleles with three repeats (most common) and four or five repeats are more common than others (10). Interestingly, bioinformatics studies for sequence alignment have shown that the allele with three repeats in this VNTR is specific to human lineage and may play a role in cognitive abilities, although the allele with two repeats is highly conserved throughout the primate lineage (19).

The incidence of the colon and gastric cancers has significantly increased over the past decade in Iran due to changes in lifestyle and diet and both cancers are one of the medical challenges in Iran (24, 25). In this study, for the first time based on our knowledge, we showed a significant association of VNTR rs58335419 located in miR-137 gene with the risk of colon and gastric cancers in an Iranian population.

In our study, we identified 12 different genotypes, most of which have a low frequency in patients or control groups in colon and gastric cancers. However, genotypes 3/3, 3/4, and 3/5 have the highest frequencies in patients and control groups in colon and gastric cancers which was consistent with previous studies (20).

The results of our study showed that genotype 3/5 significantly increases the risk of colon cancer, but none of the genotypes show a significant association with the risk of gastric cancer. However, if all genotypes are classified into one group (even rare genotypes), they can significantly increase the risk of both cancers compared to genotype 3/3. To date, the association between this VNTR polymorphism and the risk of colon and gastric cancers has not been investigated.

Consistent with our results, Mahmoudi et al. in 2020 showed that allele with 4 repeats increase the risk of a type of schizophrenia (10). However, in 2019, Pacheco et al. reported that there was no association between the length of this VNTR and Schizophrenia (19).

Given that longer VNTR length is associated with lower expression of mature miR-137, as well as decreased MIR137 in colon and gastric cancers, it seems reasonable to increase the risk of colon and gastric cancers by increasing the length of VNTR in the MIR137 gene.

Some potential limitations have been attributed to this study. First, the sample size in this study was small, therefore, the strength of the results may be affected. It is suggested that the results be verified in larger population in the future. Second, controls were randomly selected and may include some uncontrolled subjects with asymptomatic adenomatous polyps (a precursor to colon cancer).

## Conclusion

This study shows a statistically significant associations of alleles with more than three repeats in the VNTR rs58335419 of miRNA-137 gene with the risk of colon and gastric cancers in the Iranian population. Due to the longer VNTR is associated with lower expression of mature miR-137 as well as decreased MIR137 in colon and gastric cancers, it extends the importance of our findings. This may help expand the ability to develop a biomarker in colon and gastric cancers screening.

## Data Availability

The original contributions presented in the study are included in the article, further inquiries can be directed to the corresponding author.
